# *EAAC1* Gene Deletion Increases Neuronal Death and Blood Brain Barrier Disruption after Transient Cerebral Ischemia in Female Mice

**DOI:** 10.3390/ijms151119444

**Published:** 2014-10-27

**Authors:** Bo Young Choi, Jin Hee Kim, Hyun Jung Kim, Bo Eun Lee, In Yeol Kim, Min Sohn, Sang Won Suh

**Affiliations:** 1 Department of Physiology, Hallym University, College of Medicine, Chuncheon 200-702, Korea; E-Mails: bychoi@hallym.ac.kr (B.Y.C.); fate0710@hallym.ac.kr (J.H.K.); missgabong@hallym.ac.kr (H.J.K.); supsock1126@naver.com (B.E.L.); inyeol@hallym.ac.kr (I.Y.K.); 2 Department of Nursing, Inha University, Incheon 402-751, Korea; E-Mail: sohnmin@inha.ac.kr

**Keywords:** *EAAC1*, cysteine, blood–brain barrier, ischemia, glutathione, vessel disorganization

## Abstract

*EAAC1* is important in modulating brain ischemic tolerance. Mice lacking *EAAC1* exhibit increased susceptibility to neuronal oxidative stress in mice after transient cerebral ischemia. *EAAC1* was first described as a glutamate transporter but later recognized to also function as a cysteine transporter in neurons. *EAAC1*-mediated transport of cysteine into neurons contributes to neuronal antioxidant function by providing cysteine substrates for glutathione synthesis. Here we evaluated the effects of *EAAC1* gene deletion on hippocampal blood vessel disorganization after transient cerebral ischemia. *EAAC1^−/−^* female mice subjected to transient cerebral ischemia by common carotid artery occlusion for 30 min exhibited twice as much hippocampal neuronal death compared to wild-type female mice as well as increased reduction of neuronal glutathione, blood–brain barrier (BBB) disruption and vessel disorganization. Pre-treatment of *N*-acetyl cysteine, a membrane-permeant cysteine prodrug, increased basal glutathione levels in the *EAAC1^−/−^* female mice and reduced ischemic neuronal death, BBB disruption and vessel disorganization. These findings suggest that cysteine uptake by *EAAC1* is important for neuronal antioxidant function under ischemic conditions.

## 1. Introduction

Ischemic stroke is the most prevalent neurological disease and the major cause of long-term disability in adults. Cerebral ischemia is accompanied by vascular permeability changes and by fulminant cellular inflammatory processes in the brain that contribute to neuronal death after stroke and several studies have suggested that disintegration of blood–brain barrier (BBB) after ischemic insults can exacerbate ischemia-induced neuron death. A tight interplay between the brain and the peripheral immune system exists via the blood–brain barrier and this relationship leads to a vulnerability of the brain to immune response during and after stroke consisting of an early activation of peripheral immune cells with massive production of proinflammatory cytokines [1,2,3]. In addition, ischemic stroke-induced neuronal death is mostly attributed to dysfunction in the homeostasis of glutamate. Glutamate acts as the primary excitatory neurotransmitter under physiological conditions [4]. However, increased extracellular glutamate levels promote activation of the NMDA receptor causing an influx of calcium, the production of reactive oxygen species (ROS), mitochondria dysfunction and the generation of reactive nitrogen species (RNS), all of which contribute to cell death [5,6].

*EAAC1* (excitatory amino acid carrier type 1) was originally believed to function exclusively as a neuronal high-affinity glutamate transporter [7]. However, several studies later showed that *EAAC1* has a negligible effect on glutamate clearance from the extracellular space, and that this function is performed primarily instead by the astrocyte glutamate transporters, GLT1 and GLAST [8,9]. Recent studies indicated that *EAAC1* also functions as a high-affinity cysteine transporter in neurons [10,11,12,13]. *EAAC1* transport of cysteine into neurons contributes to antioxidant function by providing cysteine substrates for glutathione (GSH) synthesis [14]. Glutathione is the most abundant intracellular non-protein thiol and plays an important role in antioxidant defense [15,16,17]. When intracellular cysteine and GSH levels decrease, as is seen following brain insult, levels of enhanced oxidative stress are seen in postsynaptic neurons. Aoyama *et al.* demonstrated that *EAAC1* gene deletion leads to decreased neuronal GSH concentrations and increased oxidative injury in aged mice [18]. Our previous studies demonstrated that *EAAC1* gene deletion increased neuronal death in the hippocampus and cerebral cortex after global ischemia [19,20]. Li *et al.* also demonstrated that *EAAC1* gene deletion mice showed increased infarct volume after focal cerebral ischemia [21]. While these studies demonstrate that *EAAC1* gene deletion leads to increased oxidative injury and neuronal death after ischemia, whether blood brain barrier disruption is also involved in the process of neuronal death in *EAAC1* gene deletion mice after ischemia is untested. Thus, the present study sought to investigate the effects of limited neuronal cysteine transport due to *EAAC1* gene deletion on blood–brain barrier disruption as well as the potential relationship between oxidative neuronal injury and any observed increases in blood–brain barrier permeability and subsequent neutrophil infiltration into vulnerable brain areas.

## 2. Results

### 2.1. Ischemia-Induced Neuronal Death Is Increased in EAAC1^−/−^ Female Mice

**Figure 1:**
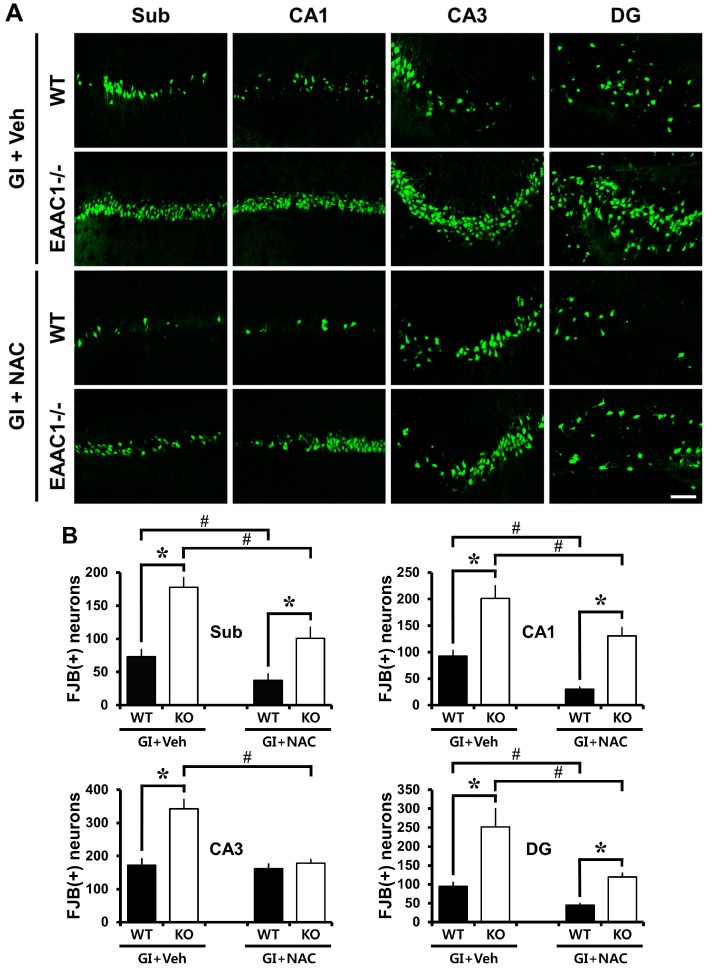
*N*-Acetylcysteine (NAC) prevents ischemia-induced neuronal death in *EAAC1^−/−^* female mice. Neuronal death was detected by Fluoro Jade-B staining. (**A**) *EAAC1^−/−^* female mice show more neuronal death than wild-type female mice after ischemia. Fluorescence images show neuronal death in hippocampal subiculum (Sub), CA1, CA3 and dentate gyrus (DG) at 3 days after transient global ischemia. Widespread Fluoro-Jade B (FJB)-positive neurons appeared in ischemia experienced wild-type (WT) mice. The number of FJB-positive neurons was significantly increased in *EAAC1^−/−^* mice. NAC treatment reduced neuron death (FJB (+) neurons) in the hippocampal subiculum (Sub), CA1, CA3 and dentate gyrus (DG) either in wild-type or in *EAAC1^−/−^* mice 3 days after ischemia. Scale bar = 100 μm; (**B**) Bar graph shows the quantified neuronal degeneration in hippocampal Sub, CA1, CA3 and DG from wild-type and *EAAC1^−/−^* mice. Data are mean + S.E.M.; *n* = 8–10, #, * *p* < 0.05.

To determine whether ischemia-induced neuronal death was aggravated by *EAAC1* gene deletion, wild-type and *EAAC1^−/−^* female mice were subjected to bilateral common carotid artery occlusion for 30 min and neuronal death was assessed by Fluoro-Jade B staining at 3 days after ischemia. Thirty minutes of ischemia induced substantial neuronal death in the hippocampus. The pattern of neuronal death is similar but the degree of neuronal death in the wild-type female mice showed less neuron death compared to male mice [19,22]. The number of degenerating hippocampal neurons was significantly higher in the *EAAC1^−/−^* female mice in the subiculum, CA1, CA3 and dentate gyrus than wild-type female mice (Figure 1).

### 2.2. NAC Prevents Ischemia-Induced Neuronal Death in EAAC1^−/−^ Female Mice

Mice were treated with either NAC or vehicle for 4 days prior to ischemia. Mice brains were harvested 3 days after ischemia. NAC pre-treatment decreased ischemia-induced neuron death in the subiculum, CA1, CA3 and dentate gyrus in both wild-type and *EAAC1^−/−^* mice (Figure 1).

### 2.3. Ischemia-Induced Neuronal Glutathione (GSH) Loss Is Increased in EAAC1^−/−^ Female Mice

**Figure 2:**
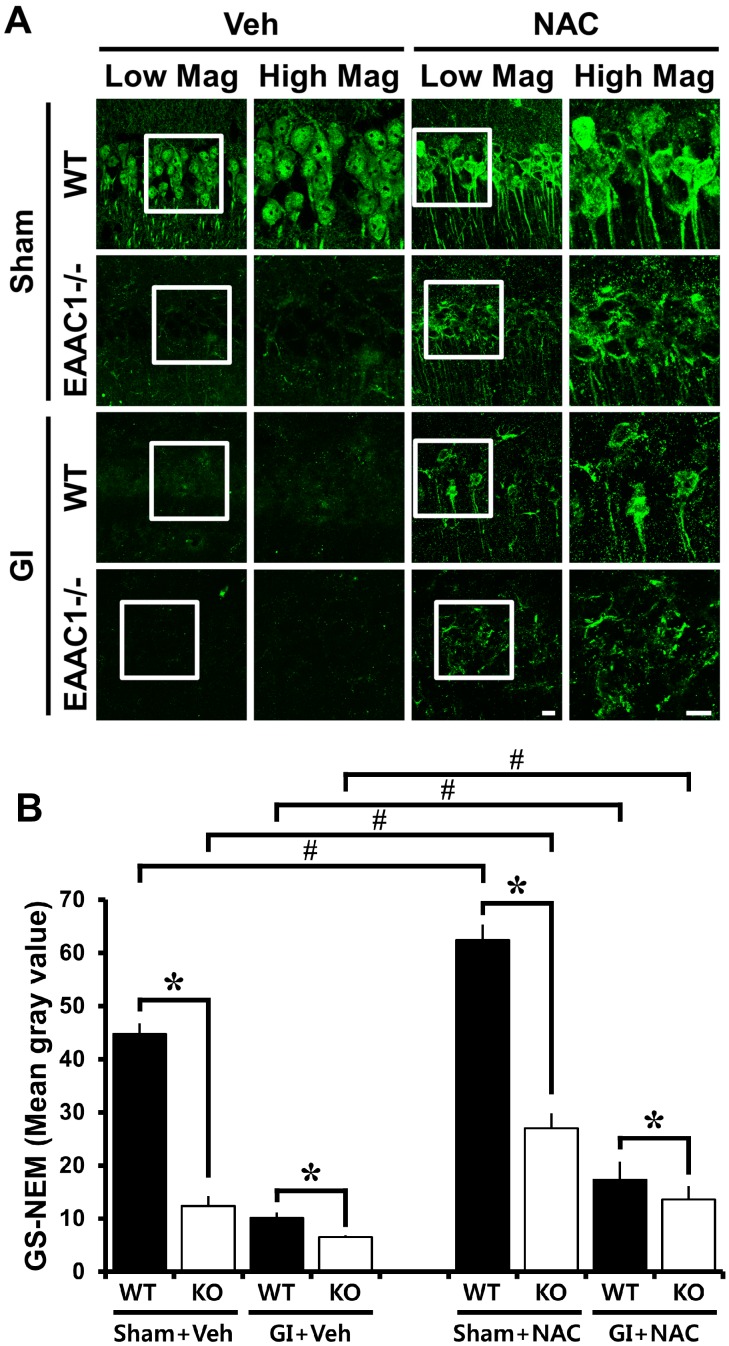
Neuronal glutathione deficiency in *EAAC1^−/−^* mice is reversed by NAC treatment. Neuronal glutathione (GSH) was detected by GS-NEM immunofluorescence staining. (**A**) There was reduced fluorescence intensity in the neurons of *EAAC1^−/−^* hippocampal sections compared to those in the wild-type slices. Ischemia-induced neuronal GSH reduction in the hippocampal CA1 is apparent in both wild-type (WT) and *EAAC1^−/−^* mice. *EAAC1^−/−^* mice show substantially increased neuronal GSH reduction compared to wild-type mice either after ischemia or after sham operation. This intensity was increased in hippocampal sections from mice treated with the cell-permeant cysteine precursor NAC in both wild-type and *EAAC1^−/−^* mice after ischemia and sham operation. Scale bar = 10 μm; (**B**) Quantification of neuronal GSH was performed from hippocampal CA1 pyramidal area. Bar graph represents mean gray value of GS-NEM fluorescent intensity. As shown in the images, neuronal GSH loss is aggravated in *EAAC1^−/−^* mice. Data are mean + S.E.M.; *n* = 3, #, * *p* < 0.05.

To test whether *EAAC1* gene deletion affected neuronal GSH content, brain sections were histologically evaluated by probing for GSH–*N*-ethylmaleimide (NEM) adducts at 3 days after ischemia. In the sham-operated animals, wild-type mice displayed many GS-NEM (+) neurons in the hippocampal CA1. However, *EAAC1^−/−^* mice showed reduced GS-NEM immunoreactivity in the CA1. Mice subjected to transient cerebral ischemia by common carotid artery occlusion for 30 min further decreased GS-NEM fluorescent intensity. In addition to that, *EAAC1^−/−^* mice subjected to ischemia showed a significant loss of GS-NEM intensity as compared to wild-type mice (Figure 2).

### 2.4. NAC Up-Regulates GSH Content in EAAC1^−/−^ Neurons

The membrane permeant cysteine prodrug, *N*-acetylcysteine (NAC), has previously been shown to normalize thiol content in *EAAC1^−/−^* mouse neurons [18]. NAC is a small molecule that freely passes through cell membranes into cytoplasmic compartments, thereby providing cysteine to cells that lack cysteine transport [23,24]. Here we evaluated neuronal GSH content using GSH–*N*-ethylmaleimide (NEM) adducts on the free-floating coronal sections [25,26]. GS-NEM fluorescence was reduced in neurons from the hippocampal sections of *EAAC1^−/−^* mice compared to wild-type mice and this signal was up-regulated in mice given i.p. injections over 4 consecutive days (Figure 2). These results confirmed the prior report that NAC can normalize reduced neuronal thiol content (which is primarily glutathione) in *EAAC1^−/−^* neurons [18].

### 2.5. Ischemia-Induced Blood–Brain Barrier (BBB) Disruption Is Increased in EAAC1^−/−^ Female Mice

**Figure 3:**
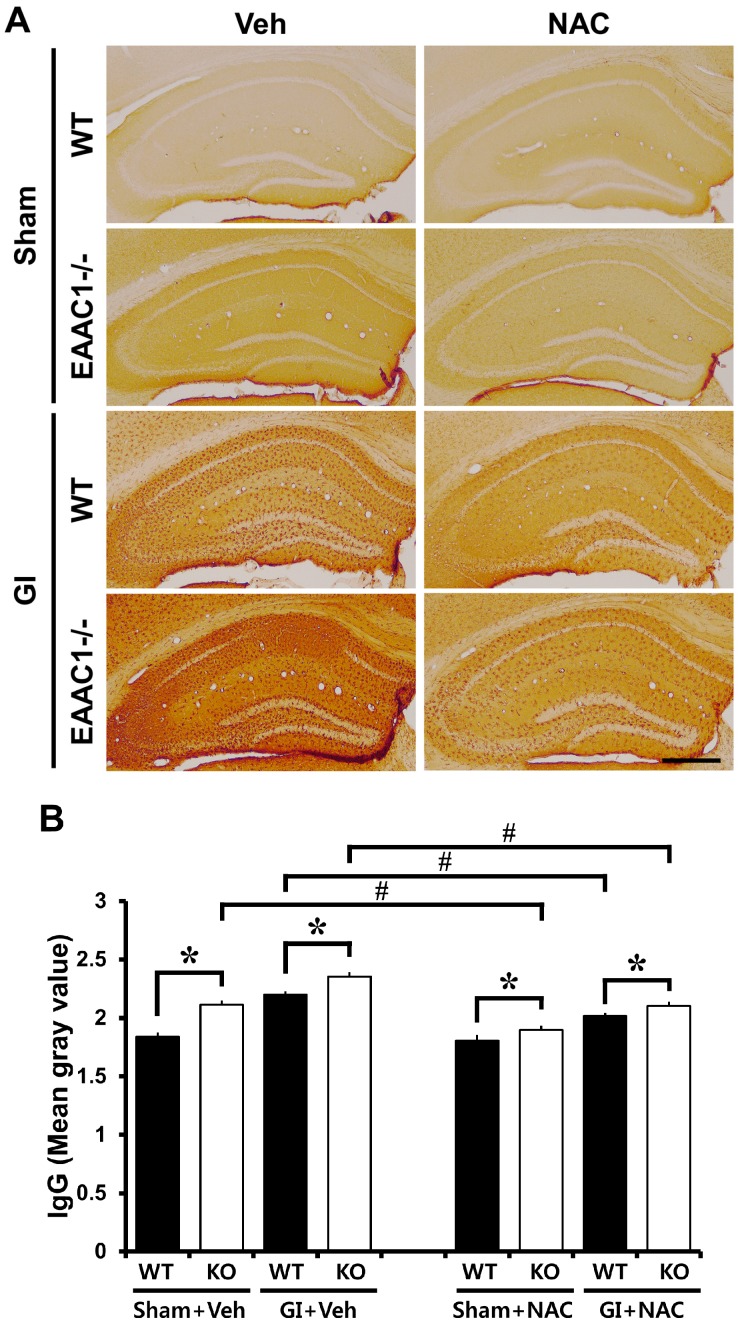
NAC reduces ischemia-induced blood–brain barrier (BBB) disruption in female *EAAC1^−/−^* mice. This figure displays blood–brain barrier damage in the hippocampus after ischemia. (**A**) Low magnification photomicrographs show IgG stained coronal hippocampal sections. In the sham-operated animals, *EAAC1^−/−^* female mice show more IgG staining in the hippocampus compared to wild-type female mice. IgG immunoreactivity was reduced in *EAAC1^−/−^* mice treated with NAC. At 3 days post-ischemia, the entire hippocampus is intensely stained with IgG-immunoreactivity indicating that substantial BBB damage has occurred. In the *EAAC1^−/−^* female mice, IgG-immunoreactivity was further increased compared to wild-type female mice. NAC treatment reduced ischemia-induced BBB damage either in wild-type or in *EAAC1^−/−^* mice. Scale bar = 200 μm; (**B**) Bar graph represents the quantification of IgG intensity in the hippocampus. The intensity is significantly different among the groups, and *post-hoc* analysis reveals the significant difference between wild-type and *EAAC1^−/−^* female mice. Data are mean + S.E.M.; *n* = 3–9, #, * *p* < 0.05.

To evaluate the putative breakdown of the BBB, we looked for a leakage of serum IgGs using immunohistochemistry [27]. In the sham-operated wild type mice, IgGs were restricted to the vessels, whereas in *EAAC1^−/−^* mice, we observed an increased diffused IgG immunoreactivity throughout the hippocampus. Immunoglobulin leakage by BBB breakdown was apparent after ischemia. There was a significant difference in IgG leakage between wild-type and *EAAC1^−/−^* female mice (Figure 3).

### 2.6. NAC Prevents Ischemia-Induced Blood–Brain Barrier (BBB) Damage

To evaluate whether NAC pre-treatment reduced breakdown of the BBB, wild type and *EAAC1^−/−^* mice were treated with NAC for 4 days prior to ischemia. NAC did not affect IgG leakage in the sham-operated wild-type mice. However, IgG immunoreactivity was reduced in *EAAC1^−/−^* mice given i.p. injections over 4 consecutive days. Pre-treatment with NAC reduced ischemia-induced IgG leakage through BBB disruption in both wild type and *EAAC1^−/−^* mice hippocampus (Figure 3).

### 2.7. Ischemia-Induced Vessel Disorganization Is Increased in EAAC1^−/−^ Female Mice

**Figure 4:**
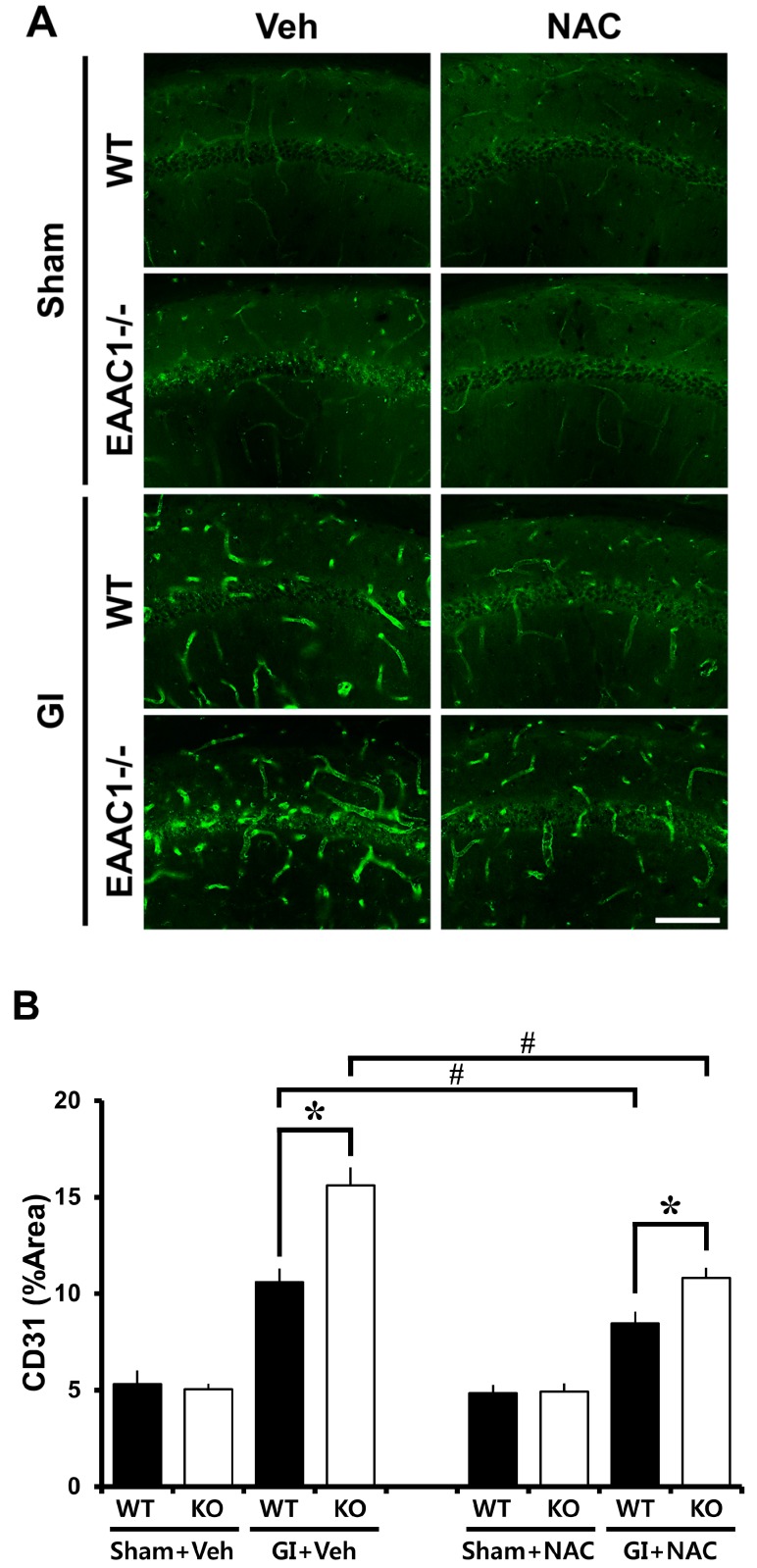
NAC reduces ischemia-induced vessel disorganization in *EAAC1^−/−^* female mice. This figure displays vessel disorganization in the hippocampus after ischemia. (**A**) Fluorescence images demonstrate vessel disorganization at 3 days after ischemia by CD31 staining. Panels show the progression of blood vessel changes in the hippocampal CA1. After ischemia, blood vessels showed increased density compared to sham-operated mice. In the *EAAC1^−/−^* female mice, density of blood vessels was further increased compared to wild-type female mice. NAC treatment reduced ischemia-induced vessel disorganization either in wild-type or in *EAAC1^−/−^* mice. Scale bar = 100 μm; (**B**) Graph represents the % area of CD31 immunoreactivity in the hippocampal CA1. Data are means + S.E.M.; *n* = 3–5, #, * *p* < 0.05.

We next examined if *EAAC1* gene deletion also induces changes in blood vessel morphology. To do so brains were histologically evaluated by CD31 immunostaining 3 days after ischemia. Particularly in the hippocampal CA1 region, the diameter and density of the blood vessels were increased after ischemia, compared to sham-operated animals. The ischemia-induced blood vessel disorganization was significantly aggravated by *EAAC1* gene deletion (Figure 4).

### 2.8. NAC Prevents Ischemia-Induced Vessel Disorganization in EAAC1^−/−^ Female Mice

Given the possibility that pre-treatment of NAC could reduce ischemia-induced vessel disorganization, wild type and *EAAC1^−/−^* mice were treated with NAC for 4 days before ischemia. NAC pre-treatment decreased ischemia-induced vessel disorganization in both wild-type and *EAAC1^−/−^* mice (Figure 4).

## 3. Discussion

The present study demonstrates that *EAAC1* gene deletion results in reduced neuronal GSH content, increased BBB disruption and increased neuronal death in female mice after transient cerebral ischemia. Pre-treatment with NAC rescued GSH content, and protected against BBB disruption and neuronal death after ischemia. These results suggest that cysteine transport through *EAAC1* is required for normal glutathione homeostasis in the brain, and that cysteine uptake by *EAAC1* is a crucial component of the brain antioxidant defense system following transient ischemia as seen in male mice [19].

Previous studies have shown that *EAAC1* can transport cysteine as efficiently as glutamate [10,28]. Cysteine uptake through *EAAC1* may be a major mechanism for supplying the substrate for glutathione synthesis in neurons. It has been shown that *EAAC1* gene deletion mice have decreased glutathione and an increased vulnerability to oxidants in hippocampal slices [18]. The deleterious effects of *EAAC1* deletion on transient cerebral ischemia also have been reported after global or focal ischemia using male mice [19,20,21]. However, none of these studies have shown the effects of *EAAC1* gene deletion on ischemia-induced brain injury in female mice.

Levels of glutathione and nitrotyrosine-containing proteins, a marker for oxidative stress, were not significantly different between the hippocampus of *EAAC1* gene deletion mice under ischemic or non-ischemic conditions. The reason for this lack of a detectable difference in the levels of glutathione and nitrotyrosine-containing proteins after injury is not clear. Glial cells may lead to an overestimation of the glutathione levels in the neurons of *EAAC1* gene deletion mice. Our previous study showed that hippocampal glutathione levels of *EAAC1* gene deletion male mice were significantly lower than those in wild-type mice [18]. However, Li *et al.* concluded that glutathione levels in the hippocampus were not different [21]. Using a newly developed probe for labeling neuronal glutathione, NEM, the present study confirmed that the neuronal glutathione level of hippocampus is in fact reduced in *EAAC1* gene deletion mice compared to wild type mice.

*EAAC1* is not known to be expressed in any blood vessels and *EAAC1* gene deletion mice and wild-type mice had similar gross cerebral vasculature. However, *EAAC1* gene deletion mice showed increased CD31 expression and IgG leakage in the hippocampus after transient cerebral ischemia, both markers for BBB disruption. NAC treatment reduced ischemia-induced CD31 expression and IgG leakage both in wild-type mice and in *EAAC1* gene deletion mice. This result demonstrates that increased oxidative stress in the brain blood vessel after ischemia is reversed by NAC treatment. Thus, these results clearly show that neuronal *EAAC1* is indirectly involved in brain ischemic tolerance.

## 4. Experimental Section

### 4.1. Ethics Statement

Animal studies were approved by the Committee on Animal Use for Research and Education at Hallym University (protocol # Hallym 2013-128, 22 January 2014), in compliance with NIH guidelines. Animal sacrifice was performed under isoflurane anesthesia and all efforts were made to minimize suffering.

### 4.2. Mouse Colony

*EAAC1*^−/−^ mice were descendent of the strain established by Peghinni *et al.* [29], in which exon 1 is disrupted by a neomycin resistance (NEO) cassette. These mice were outbred to wild-type C57/BL6 mice for more than 10 generations prior to these studies. A wild-type (WT) control colony was maintained using the wild-type offspring from the latter outcrosses. WT breeding stock and *EAAC1^−/−^* mice were intercrossed at least once every 8 generations to prevent genetic drift, in accordance with the Banbury Conference recommendations [30].

### 4.3. Transient Ischemia

Female mice from the *EAAC1^−/−^* colony (C57/BL6 strain background) were used. Wild-type female C57/BL6 mice (3–5 months old, weight 30–50 g) from DBL (Chungcheongbukdo, Korea) were used as controls. Wild-type or *EAAC1^−/−^* mice were anesthetized with 2% isoflurane in a 75:25 mixture of nitrous oxide and oxygen. Both carotid arteries were exposed through a midline neck incision. Both common carotid arteries were loosely encircled with a 4/O silk suture before the start of the occlusion. Small aneurismal clips were applied to both common carotid arteries occlusion. Wild-type or *EAAC1^−/−^* mice were subjected to bilateral common carotid artery occlusion for 30 min while anesthetized with 1% isoflurane [19,20,22]. At the end of the 30 min ischemic period the aneurismal clips were removed and the common carotids were inspected for normal recovery of blood flow. Following suture of the skin incision, anesthetics were discontinued. When mice showed spontaneous respiration they were returned to a recovery room maintained at 37 °C. Core temperature was kept at 36.5–37.5 °C with a homoeothermic blanket control unit (Harvard apparatus, Holliston, MA, USA). Sham operated animals received the same neck skin incision under isoflurane anesthesia but carotid occlusion was not performed.

### 4.4. Neuron Death

Neuronal death after transient cerebral ischemia was evaluated after a 3-day survival period. Mice brains were perfused with 4% paraformaldehyde (PFA) after 0.9% saline perfusion. After overnight post-fixation, brains were submerged in 30% sucrose for cryo-protection until they sank to the bottom. Five coronal sections were collected from each brain, spaced 80 µm apart and spanning the hippocampus. Brain sections were stained by the Fluoro-Jade B method (Histo-Chem, Jefferson, AR, USA) [31]. A blinded observer counted the total number of Fluoro-Jade B-positive neurons in each structure of interest, in both hemispheres [32]. Data from each animal were expressed as the mean number of degenerating neurons per structure of interest.

### 4.5. Detection of Reduced GSH

To detect the reduced form of GSH in the brain sections, we probed for GSH–*N*-ethylmaleimide (NEM) adducts on the free-floating coronal sections [25,26,33]. At the indicated time points, mice were deeply anesthetized and transcardially perfused with 0.9% saline followed by 4% paraformaldehyde (PFA). The harvested brains were post-fixed for one hour with 4% PFA and transferred to 30% sucrose for cryo-protection. Thereafter, the entire brain was frozen and sectioned with a cryostat at 30 μm thickness. Brain sections were incubated with 10 mM NEM for 4 h at 4 °C, washed, and incubated with mouse anti-GS-NEM (diluted 1:100, Millipore, Billerica, MA, USA). After washing, the sections were incubated with Alexa Fluor 488-conjugated goat anti-mouse IgG (diluted 1:250, Invitrogen, Carlsbad, CA, USA) for 2 h. The sections were mounted and photographed with a Zeiss confocal microscope. To quantify GSH intensity, individual neurons from the brain section images were selected as regions of interest (ROIs) and measured using Image J (NIH, Bethesda, MD, USA). Briefly, to quantify the GSH intensity, the image was loaded into Image J version 1.47c and converted into an 8-bit image through the menu option Image→Type→8-bit. Then, regions comprising individual neurons in the CA1 image were selected as ROIs. The resulting image was then binarized and restricted to the region of measurement for individual neurons. To measure this area, the menu option Analyze→Measure was selected and then the signal from individual neurons was expressed as mean gray value [33].

### 4.6. Detection of Blood–Brain Barrier Disruption by IgG Immunostaining

Mice brains were examined for the extravasation of presumed endogenous serum IgG after ischemia [27,34,35]. Mice were sacrificed 3 days after the ischemia. The avidin–biotin–peroxidase complex (ABC) immunoperoxidase method was employed to detect IgG-like immunoreactivity [36]. Brains were fixed by perfusion (4% PFA). Coronal sections (30 μm thick) were incubated with chicken serum, followed with purified biotinylated horse anti-mouse IgG (Vector Laboratories, Burlingame, CA, USA) at a dilution of 1:250. IgG immunoreactivity was quantified by using Image J and expressed as mean gray value [37].

### 4.7. Detection of Vessel Disorganization

For immunohistochemical staining, monoclonal rat anti-mouse CD31 (diluted 1:50, BD Pharmingen, Franklin Lakes, NJ, USA), diluted in PBS containing 0.3% Triton X-100, were used as the primary antibodies and incubated overnight at 4 °C. The sections were washed three times for ten minutes each with PBS, incubated in Alexa Fluor 488-conjugated goat anti-rat IgG (diluted 1:250, Invitrogen) for 2 h in the same solution as the primary antiserum. The sections were mounted and photographed with a fluorescence microscope (Olympus upright microscope IX70, Olympus, Tokyo, Japan). CD31 immunoreactivity was quantified using Image J and expressed as % area.

### 4.8. N-Acetylcysteine (NAC) Administration

NAC was dissolved with 0.9% saline and was injected into the peritoneal space (150 mg/kg, Sigma–Aldrich, St. Louis, MO, USA) once per day for 4 consecutive days in wild-type and *EAAC1^−/−^* mice. Control mice were injected with saline only (vehicle). After 4 days of NAC injection, mice were subjected to transient cerebral ischemia on day 5. No NAC was injected into mice on the day that ischemia was performed [20].

### 4.9. Confocal Microscopy

Fluorescence signals were detected using a Zeiss LSM 780 confocal imaging system (Zeiss, New York, NY, USA) with a sequential scanning mode for Alexa 488. Stacks of images (1024 × 1024 pixels) from consecutive slices of 0.66–0.7 μm in thickness were obtained by averaging eleven scans per slice and were processed with ZEN 2010 (Zeiss).

### 4.10. Statistical Analyses

All data were expressed as the mean ± S.E.M. Statistical significance was assessed by analysis of variance (ANOVA) followed by the Student–Newman–Keuls *post-hoc* test comparing all groups.

## 5. Conclusions

Prior studies have shown that ischemia-induced brain injury is aggravated by *EAAC1* gene deletion. This increased brain injury is believed to be a result of reduced neuronal antioxidant function. In the present study we found that female mice lacking *EAAC1* also exhibit reduced neuronal GSH content and increased oxidative stress after ischemia, as seen in male mice. The present results show that deletion of the neuronal cysteine transporter, *EAAC1*, not only increased neuronal death but also increased BBB disruption after ischemia. Thus, the present study suggests that cysteine transport through *EAAC1* is required for normal homeostasis and neuron survival after ischemia in the brain, which can be reversed or improved by prior NAC administration.

It should be noted, however, that in our studies we have been using fully cycling female animals without measuring circulating estrogen levels, therefore, neuroprotection by estrogens may have contributed to the obtained results. In future studies we will address this issue by also employing ovariectomized animals and measuring estrogen levels in the experimental animals.
